# Gunpowder Stains

**Published:** 1892-01

**Authors:** 


### GUNPOWDER STAINS.

These may be removed by painting with biniodide of ammonium, distilled water, equal parts; then with dilute hydrochloric acid, to reach the tissues more deeply affected.—*Review de Therapeut.*

